# Why Do Patients Leave against Medical Advice? Reasons, Consequences, Prevention, and Interventions

**DOI:** 10.3390/healthcare9020111

**Published:** 2021-01-21

**Authors:** Asseel Albayati, Steven Douedi, Abbas Alshami, Mohammad A. Hossain, Shuvendu Sen, Vito Buccellato, Anamarrie Cutroneo, Jason Beelitz, Arif Asif

**Affiliations:** Department of Medicine, Jersey Shore University Medical Center, Hackensack Meridian Health, Neptune, NJ 07753, USA; Asseel.Albayati@hackensackmeridian.org (A.A.); Abbas.Alshami@hackensackmeridian.org (A.A.); Mohammad.Hossain@hackensackmeridian.org (M.A.H.); Shuvendu.Sen@hackensackmeridian.org (S.S.); Vito.Buccellato@hackensackmeridian.org (V.B.); Anamarrie.Cutroneo@hackensackmeridian.org (A.C.); Jason.Beelitz@hackensackmeridian.org (J.B.); Arif.Asif@hackensackmeridian.org (A.A.)

**Keywords:** discharge, quality improvement, against medical advice, AMA, DAMA

## Abstract

Background: A patient decides to leave the hospital against medical advice. Is this an erratic eccentric behavior of the patient, or a gap in the quality of care provided by the hospital? With a significant and increasing prevalence of up to 1–2% of all hospital admissions, leaving against medical advice affects both the patient and the healthcare provider. We hereby explore this persistent problem in the healthcare system. We searched Medline and PubMed within the last 10 years, using the keywords “discharge against medical advice,” “DAMA,” “leave against medical advice,” and “AMA.” We retrospectively reviewed 49 articles in our project. Ishikawa fishbone root cause analysis (RCA) was employed to explore reasons for leaving against medical advice (AMA). This report presents the results of the RCA and highlights the consequences of discharge against medical advice (DAMA). In addition, the article explores preventive strategies, as well as interventions to ameliorate leaving AMA.

## 1. Introduction

Discharge against medical advice (DAMA) is defined as when a patient chooses to leave a hospital before the healthcare team recommends discharge from the hospital [1]. With a significant and increasing prevalence of up to 1–2% of all hospital admissions, leaving against medical advice (AMA) affects both the patient and the healthcare provider [2,3]. This action leaves the patient with inadequately treated medical problems and increased risk for readmissions [1,4]. Even though no hospital is willing to allow DAMA due to its adverse consequences, this issue has become one of the most common problems in our current healthcare system. Choi et al., in a retrospective matched cohort study of 656 patients, found that the risk of readmission was 12 times more in patients who leave AMA when compared to the non-AMA group [5]. They also found that the AMA group had an increased 12-month all-cause mortality (6.7% vs. 2.4%, *p* = 0.01). Though few data are available on the estimated total costs to the healthcare system, the increased mortality and rate of readmission to hospitals certainly impose an economic burden [1]. All the while, the treating physician may be struggling to fulfill the pledge they took to benefit the patient and respect their wishes [1]. This problem affects both patients and physicians, but there may be solutions to this prevalent issue. In our study, we explore the risk factors, demographics, perspectives of patients and the healthcare system, consequences, prevention strategies, and other factors to tackle discharges AMA.

## 2. Materials and Methods

We searched published studies indexed in the Medline database using the PubMed interface relevant to the scope of this literature review. We used the keywords “discharge against medical advice,” “DAMA,” “leave against medical advice,” and “AMA.” The search was limited to studies published in English. We aimed to select studies published during the last 10 years; however, this was not a strict selection criterion. To approach this problem comprehensively, we conducted a root cause analysis (RCA), which is a tool that has been commonly utilized in healthcare systems to identify the potential causes of medical errors and develop improvement strategies for the quality of care [6]. There are different techniques to conduct RCA. An Ishikawa, or fishbone, diagram is frequently used in the quality-improvement framework [7]. The final selection of articles was made by agreement between the authors.

**Figure 1:**
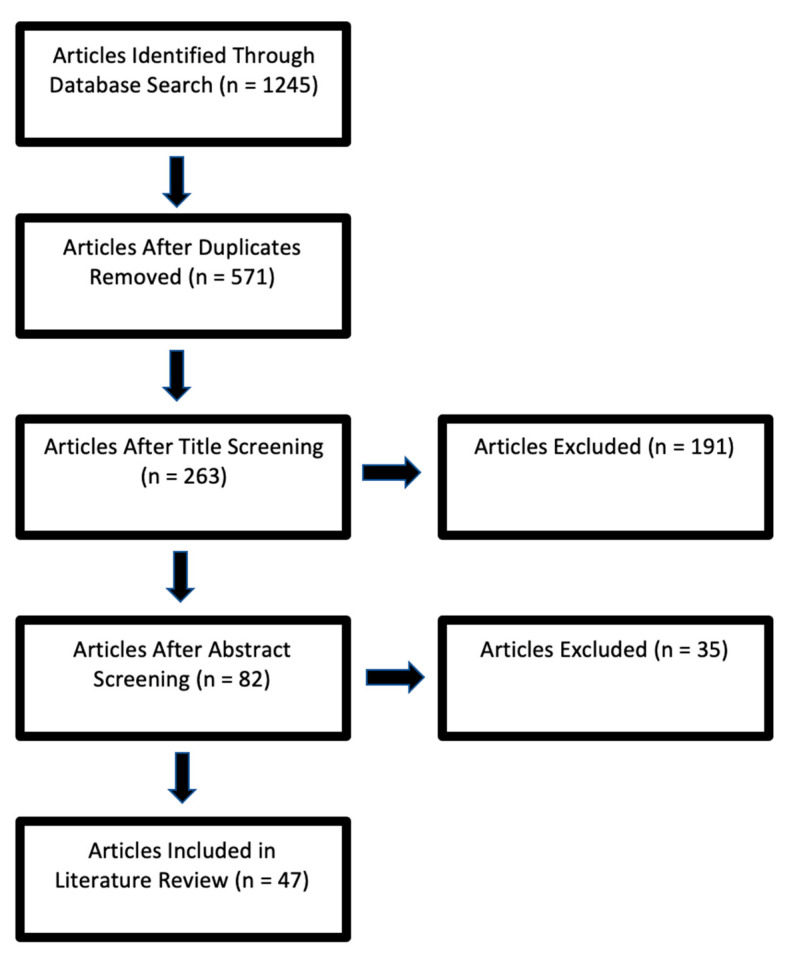
Flowchart for inclusion of articles in the literature review.

**Figure 2:**
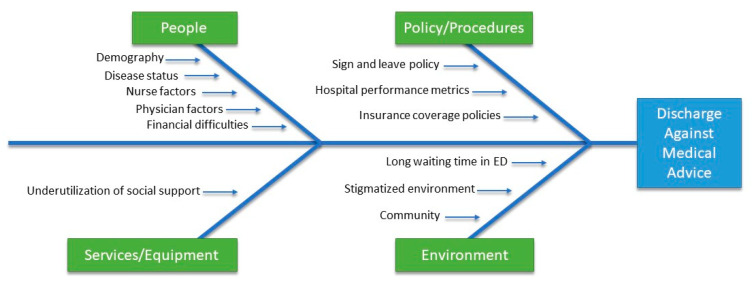
A fishbone diagram showing the causes and categories for discharges against medical advice.

Of the 1245 studies meeting our selection criteria, 49 studies were selected to be included in this literature review (Figure 1). According to a report published in 2012 by the Healthcare Cost and Utilization Project, there was a 41% increase in discharges against medical advice between 1997 and 2011 [8]. Numerous factors have been reported that increase the likelihood of patients leaving AMA, such as male gender, early age, lack of health insurance, substance abuse, alcohol-related disorder, mental-health problems, unhappiness with the provided care, financial constraints, lack of clinical improvement with the received treatment, dissatisfaction with the hospital environment, discontentment with the behavior of the medical staff, social circumstances, absence of advanced medical services, lack of coordination of care, and length of stay [3,4,9,10,11,12,13,14]. We categorized these causes into four categories: people, policy/procedures, environment, and services/equipment (Figure 2).

## 3. Discussion

The patients, who choose to be admitted to the hospital, are implicitly consenting to an unwritten contract between the patient and the healthcare provider. This “virtual contract” first entails taking all the measures to improve the patient’s health, despite any business the patient might need to attend to outside the hospital. Second, the patient will remain in the hospital as long as it is necessary to achieve the goal that is determined by the patient and the physician, but no longer than that. Based on the knowledge and skills of the physician that are trusted by the patient, a decision is to be taken to discharge the patient from the hospital [15]. However, some patients, driven by different factors, opt to breach this contract and leave the hospital prematurely.

### 3.1. Reasons

#### 3.1.1. People Factors

##### Physicians’ Factors

Patients may leave AMA because they disagree with the clinical judgment of their physicians about their medical status. A more serious reason is a conflict between the caregiver and the patient.

One of the prime areas of concern has been the lack of coordination among healthcare providers. Branched out into various subspecialties, patient care has become the denouement of multiple, often disjointed forces of medical management. While it results in overutilization of resources, extended length of stay, and increased financial burden, the biggest fallout has been failing to mete out coercive, comprehensive, coordinated care. The ensuing lack of clarity in patient care is logically and strongly reflected in the patient’s own lack of understanding of a treatment plan that he or she is subjected to. Subsequent patient dissatisfaction and desire to leave the hospital due to unmet needs become natural consequences.

Other reasons include a general mistrust in the entire system that some patients develop before coming to the hospital. This can be triggered by a bad experience of the patient or a family member with the provider. Pain control is very challenging, especially among patients with a history of substance abuse. This will potentially compromise the patient-physician relationship, and will increase the patient’s chance of leaving against medical advice due to inadequate treatment [10,16,17,18,19].

Other reported reasons that contributed to a patient deciding to leave the hospital against medical advice include poor customer service, respect, and quality of care. This perception was determined majorly by the cultural background and the healthcare team [15].

#### 3.1.2. Nurses’ Factors

Most of the time, nurses are the first to identify the patients who are willing to leave against medical advice. Nurses’ contribution to the development of new strategies is of utmost importance, especially in recognizing the early triggers that lead the patients to decide to leave. Collaboration with nurses to design new interventions that aim to reduce DAMA is needed. A team-based approach with a shared goal of early recognition and efficient intervention can address the trigger to leave, and find the solution to achieving lower rates of patients leaving against medical advice [20,21].

#### 3.1.3. Demographic Characteristics

A review of many studies concluded that being an African-American male, being of young age (<40 years), lacking medical insurance or having noncommercial health insurance, and a low household income (<$30,000/year) are decisive factors that predict being discharged against medical advice within a few days of admission (*p* < 0.01) [3,15,22,23].

Of note, Franks and Fiscella et al. studied an extensive psychiatric database from California, Florida, and New York in 2007. They noticed that there was no association between race/ethnicity and discharges against medical advice after adjusting for the individual and socioeconomic variables [15]. They hypothesized that African Americans are more inclined to leave against medical advice as they feel more than others; they are disrespected and receive unfair treatment by the healthcare system. On the other hand, the lower rates of AMA among Hispanics and other ethnic minorities were believed to be because they are culturally more accepting of the healthcare system [15].

#### 3.1.4. Associated Diseases

Data have shown that past medical history is another significant risk factor in predicting who is going to leave against medical advice. DAMA was most frequently reported in patients with a history of substance abuse (23%); this includes alcohol abuse and/or intravenous drug abuse (IVDU, i.e., people who inject drugs PWID). Other conditions that contribute to high odds of leaving AMA include psychiatric diagnoses (20%), HIV infection (13%), heart failure (4.9%), asthma (4%), and others [13,24].

Substance abuse: A retrospective study of 234,141 patients worldwide reported a significant correlation between the incidence of DAMA and patients who are affected by substance abuse, with a mean ranging from 20% to 54% (*p* < 0.001). DAMA in patients with substance abuse was reported for 20% to more than 50% of them [25]. In a systematic analysis, the mean age of DAMA before 2000 was around 35 years old; however, after 2000, studies showed a mean age of DAMA around 58 years old. [12].

Psychiatric illness: Over the years, mental illnesses have been defined by legal and societal pressures. There have been many advances to facilitate healthcare to reach to these patients. Legal decisions encouraged mental health system access; many humanistic social movements emerged, such as the community support system movement. Those movements encouraged patients with psychiatric illness to take an active role in the course of their treatments. Moreover, government policy made outpatient and community services available to patients. This change in the dynamics of handling psychiatric illnesses led to both a positive and negative impact on inpatient psychiatry. This empowerment for the patients gave them the right to terminate the management plan and eventually limit the options of treatment once they decided to step outside the hospital [26].

This is profoundly noticed among young single men with psychiatric conditions and comorbid diagnoses of personality or substance-use disorders. This group of patients was found to have a pessimistic attitude toward treatment from the beginning, and they often engage in antisocial, aggressive, or disruptive behaviors, ending with discharge against medical advice [26].

Human immunodeficiency virus disease: Significant rates of discharge against medical advice have been seen among patients who have a past medical history of HIV or AIDS-related diseases. Gradually, after the discovery of antiretroviral therapy in the late 1990s, higher rates of HIV patients have been admitted for reasons other than the disease pathogenesis itself [27,28,29]. From 10–30% of HIV patients are discharged against medical advice. These HIV patients happen to share admission reasons related to treatment non-adherence, poor social support, and difficulty establishing or maintaining access to care [5,23,30]. Anis. H.A.H. et al. conducted a study of 981 patients admitted for HIV/AIDS. The results showed that patients left AMA on the day of welfare checks in 13% (125 patients), and most of them were readmitted within 30 days of leaving against medical advice [23].

A Canadian study in 2002 showed a correlation of DAMA among HIV/AIDS patients. Of 981 patients with HIV/AIDS, 13% left AMA (125). Compared to the patients who were formally discharged, it was found that those who left AMA were readmitted with a related diagnosis in the next 30 days (odds ratio 5.00, 95% CI 3.04–8.24). Moreover, they had a longer length of stay in the follow-up visits [19,23].

Heart failure: With a prevalence of 4%, the most common reason for DAMA among this population was related to reluctance to undergo an invasive therapeutic procedure (revascularization) and decided to leave AMA. The most frequent reason for refusing surgical intervention was the patient’s fear of the procedure [13].

Asthma: DAMA among asthma admissions followed the same demographics of other DAMA patients: being young, male, low-income, and more likely to be uninsured or have Medicaid insurance. The severity of the disease was not related to the rates of DAMA in this study population. Moreover, a high number of patients who left AMA were twice as likely to require admission to the ICU and require intubation. Those were also admitted through the emergency department [31].

Sickle-cell disease: Although sickle-cell disease patients (SCC) hold a potential reason for pain-management issues and disease recurrence, this has not been studied on a large scale [3]. It has been reported that this category has a 2.4% risk of leaving AMA. Moreover, the readmission risk among SCC at 7-days and 30-days was 2.9 and 1.8 times, respectively.

#### 3.1.5. Financial Burden

A study conducted in 2019 in a tertiary care center explored the reasons behind discharges against medical advice. It was found that 40.6% of the patients who left AMA had a lack of finances. The same study also reported other significant causes, including domestic problems (18.2%), a wish to continue treatment elsewhere (9.8%), dissatisfaction with healthcare (7.9%), dissatisfaction with physical arrangements (2.3%), dissatisfaction with ward routine [0.7%], and denial by payer (0.5%). Of those studied, 4.9% reported other reasons, among which was ”feeling better” [32].

Domestic problems were the second reason reported in this study; these entail the need to take care of dependent children or a spouse, or reasons not specified (15.2%) [33]. In the same study, among those who left AMA, 367 (85.5%) patients reported that they have to leave because they were self-paying, while 41 patients (9.6%) reported being paid from a third party, and 21 (4.9%) patients were paid by the employer [32].

### 3.2. Services/Equipment Factors

#### Underutilization of Social Support

It is crucial to address the reasons and concerns that make a patient decide to interrupt the management plan and leave the hospital prematurely. Addressing these concerns becomes challenging once a patient decides to leave AMA. Early recognition of these concerns can help build trust and scrutinize the patient-health provider relationship.

In a marginal population, as soon as a patient feels an improvement in their health, they will try to prioritize other aspects of their lives. Addressing those aspects using a social-support service while in the hospital has helped to persuade patients to finish their management plan, and prevented them leaving against medical advice. For instance, social support has been provided to address a pet at home that needed care [33].

Another example of services that can help prevent leaving against medical advice is inpatient detoxification or residential services and the use of buprenorphine or methadone. It was found that inpatient prescription of methadone leads to a 50% reduction of the chances that an intravenous-drug-user patient will leave AMA [33]. Those who received inpatient buprenorphine or methadone had a 76% overdose risk reduction by their 12-month follow-up. Moreover, they were 32% less likely to have a serious opioid-related acute care follow-up [34].

### 3.3. Policy/Procedures Factors

#### 3.3.1. Sign-Leave Policy

The orientation of the patient regarding the expected course of inpatient treatment early during the admission to the hospital has helped improve the patient-provider relationship bond and built trust in the healthcare system. Implementation of a position for patient advocacy resulted in a significant decrease in the rates of discharge against medical advice in the study conducted by Brook M et al. That patient advocacy program enhanced the importance of the provider-patient relationship [26]. This intervention was essential to keep the patient in the hospital, and evident by recognizing that most of the patients who left the hospital against medical advice were those who were admitted on the weekends and discharged during the evening and night shifts, the periods when the hospital is short-staffed. Ibrahim SA et al. suggested another strategy to mitigate discharges against medical advice: the engagement of the patient in planning his care and involving pain specialists early during management [15].

#### 3.3.2. Hospital Performance Metric

##### Insurance Coverage Policies

Studies have shown that insurance status is part of the risk factors that make the patient leave AMA [85%], after adjusting for the effects of age, gender, race, income, and length of stay, as well as the location, size, and teaching status of the hospital [32]. Moreover, the readmission rate is higher for patients who were discharged against medical advice compared to formal discharges. This is associated with an increased cost burden on the healthcare system. This is further exacerbated by the fact that Medicare and uninsured hospitals account for half the cost of AMA patients, compared to one-quarter of other types of discharges [22]. Uninsured patients have a higher probability of leaving AMA and leaving before completing their treatment [32].

### 3.4. Environmental Factors

#### 3.4.1. Emergency Department Waiting

It has been reported by Ibrahim SA et al. that some patients leave the hospital against medical advice because of the administrative delays in the discharge processes [15]. Neurological manifestation has been linked with patients leaving against medical advice from the emergency department. The most common neurological-presenting symptoms were seizures, headaches, and sensory deficits [35]. Those between of the ages of 30–50 years old and those older than 70 years old tend to leave against medical advice from the emergency department even before being admitted to the hospital. Furthermore, the study showed that 140 patients (59.3%) were self-presenting to the hospital (*p* < 0.001). However, 75 patients (31.8%) were presented by emergency medical services (EMS) (*p* < 0.001). Seizures (41 patients, 18.6%) and sensory deficits (34 patients, 15.4%) were the most common presenting symptoms. More than half of those patients (156, 55.5%) left without conducting the physical examination, and 125 patients (44.5%) left after completing the physical examination and the diagnostic workup that led to the indication of admission. Although the reasons for DAMA were not sought for these patients, it was expressed that feelings of anger and dissatisfaction were behind leaving the hospital. It was believed that those feelings were masking other feelings of fear and hopelessness to their medical condition. One suggested strategy to reduce the rate of premature discharges is to increase awareness and proactively engage in communication guided toward better patient-physician relationships and shared decision-making regarding patient care [35].

#### 3.4.2. Community

Hospitals that serve low-income populations also have a higher index of AMA discharges [25]. Race as a factor for leaving AMA was not significant in different studies. However, a median household income of less than $20,000 and an insurance status of uninsured or Medicaid showed an increased risk for leaving against medical advice [32].

Socioeconomic status seems to play a role in the possibility of DAMA with an inverse correlation, where higher rates of DAMA were reported at a hospital serving a lower-class population (2.2%) compared to a hospital that served a primarily middle- and upper-class population (0.8%) [25]. In the United States, socioeconomic status is strongly linked to health-insurance type and eligibility for Medicaid. Weingart et al. and Jeremiah et al. reported an almost twofold increase in the likelihood of leaving AMA in patients who lacked health insurance and those who were not eligible for Medicaid [25,36]. The triad of financial solvency, possession of health insurance, and accessibility to primary care have been shown to be the fulcrum of improved healthcare. Absence of all these has been attributed to lack of education and lack of awareness, consequently leading to decisions that are irrational and self-harming [37].

### 3.5. Consequences of Leaving AMA

Almost all the studies demonstrated the same consequences for patients leaving against medical advice. These include adverse disease outcomes that led to higher readmission rates within 7-days, 30-days, and 90-days. More extended hospital stays for those who were readmitted after DAMA were also reported. Some studies reported an increase in mortality rates for those who left against medical advice versus a formal discharge from the hospital.

#### 3.5.1. Readmission

Many studies have shown that patients who leave the hospital against medical advice have a higher risk of being readmitted to the hospital soon (frequency ratio 1.25, 95% CI 1.11–1.42) [23]. In 1998, a study was conducted in Boston by Weingart SN et al. in patients who were discharged against medical advice that compared them to those who were discharged formally following medical advice. The study showed a 14% risk of readmission to the hospital within seven days of leaving AMA, compared to 7% for standard discharge [36]. Follow-up within 15 days of discharge AMA revealed that the risk of readmission increased by up to 21% (*p* < 0.001) when compared to a planned discharge (3%) [4]. Furthermore, 95% of those who were readmitted within 15 days received the same diagnosis they were admitted for in the first place [4]. By 90-day follow-up, same-diagnosis readmission was reported to be 69% [36].

The risk of readmission is more than doubled for patients leaving AMA compared to planned discharge, and that risk remains high for a long time. This was evident in the Yong TY et al. study conducted in 2013. The study demonstrated that those who decided to leave AMA have a 2.36 times higher risk of readmission to the hospital within seven days (95% confidence interval (CI), 1.99–2.81; *p* < 0.001). The risk of readmission remains high up to 28 days after DAMA, with a risk rate of 1.66 (95% CI, 1.44–1.92; *p* < 0.001). Moreover, the study showed that we should still expect readmission of those patients who left AMA up to one year after discharge, with a risk rate of 1.31 (95% CI, 1.19–1.45; *p* < 0.001) [38].

#### 3.5.2. Length of Stay

An expected consequence for patients who leave against medical advice and are readmitted to the hospital is a higher risk of staying in the hospital almost double the time they are expected to stay should they be discharged formally (2.3 days vs. 4.7 days) [22]. This resulted in a 56% higher cumulative cost of hospitalization than expected [22].

#### 3.5.3. Cost of Readmission

The financial burden is not imposed on the patient only; the healthcare system is also affected by the consequence of leaving against medical advice. Reports have shown that the cost of an average stay was USD 3716.00 for those patients who left as planned. However, patients who left against medical advice and were readmitted had an average bill of USD 10,761.56 for staying 4.7 days in the hospital upon readmission. This translates to 56% higher than if the patient were to have a planned discharge. This augmented difference is because more than half the cost of DAMA is under the responsibility of Medicaid and uninsured hospitals, compared to one-quarter of the total cost for standardized discharges [22].

For instance, the healthcare system is significantly impacted financially by sickle-cell patients who leave against medical advice. In 2017, Alfandre David et al. demonstrated the repercussions of this isolated group of patients who left the hospital prematurely. This group of patients cost hospitals USD 609 million for a total of 95,445 hospitalization days due to readmissions [3].

#### 3.5.4. Increased Mortality

The mortality risk for those who leave against medical advice is double 28 days after discharge (*p* = 0.02) [38]. Furthermore, the mortality risk is still high even after that. According to a study by Yong TY, it is 1.4 (*p* = 0.025) and 1.2 (*p* = 0.049) times for 1-year and up-to-9 years, respectively [38].

Examples of this in HIV-infected patients were found in two studies conducted by Palepu et al. in 2003 and Choi et al. in 2011, in which both concluded that HIV-infected patients who decide to leave AMA will end up having high readmission and mortality rates compared to planned-discharged patients [5,39].

### 3.6. Prevention Strategies for AMA

#### 3.6.1. At the Provider Level

Patients may opt to leave the hospital for different reasons. Understanding a patient’s reasons and trying to address them seems to be a reasonable strategy. For example, stressing the importance of blood-pressure control before discharge may not convince a patient who wants to leave due to financial difficulties. Therefore, counseling aiming to convince the patient not to leave AMA should be directed toward their needs. The central core of this kind of communication is to help identify any potential issues that can be addressed without necessarily leaving the hospital. If there is no way to address these issues, and the patient still prioritizes leaving the hospital over his health concerns, this decision should be respected [1].

Additionally, physicians should attempt to formulate a management plan that takes the patient’s aspects as a whole rather than focusing on clinical entities alone. Involving the patients in the decision-making process by addressing their extra-clinical concerns will improve their agreement with the management plan and decrease the chances of them leaving AMA. Some of these management plans might be considered suboptimal by clinicians; however, they remain acceptable alternatives in medical practice, and they are better than no management at all. For example, a patient with cellulitis who failed outpatient treatment and is refusing admission to the hospital may benefit from an intravenous antibiotic regimen at home, and adequate education on the symptoms of deterioration that would warrant coming back to the hospital would be a better plan than the patient leaving against medical advice with no treatment [3]. Physicians may argue that should the alternative plan fail, and a bad outcome occurs, they will become vulnerable to litigation. However, discharge AMA does not necessarily provide legal immunity [40]. Also, adequate documentation of the patient’s refusal of admission despite medical advice and the provision of the alternative plan, though suboptimal, was in an attempt to reduce harm, is a better strategy to protect the physician from such potential litigation. In addition, policymakers, through legislation, can better characterize the physician-patient relationship during these situations to increase the physicians’ comfort to provide alternative plans.

#### 3.6.2. At the Hospital Level

Several studies have shown that healthcare providers have negative attitudes toward patients with addiction [41,42]. In addition, patients with substance abuse reported leaving the hospital against medical advice because of the staff’s stigmatizing attitude [43]. These negative attitudes originate due to a lack of knowledge among the medical staff, and education programs focused on addiction and dealing with patients with addiction were shown to improve practitioners’ attitudes and skills to manage these patients and achieve better outcomes [44,45]. Howard and Holmshaw found that healthcare providers who received training had a better attitude towards these patients [42]. Similarly, Gerace et al. found that implementing an educational program focused on recognizing alcohol and drug-related misuse disorders, using available resources, and managing these patients’ pain, led to a significant increase in the nurses’ confidence in treating these patients and in treatment optimism [45]. Furthermore, such education programs may be implemented early in career development. Muzyk and coinvestigators created a training course that adopted the Mezirow’s transformative learning theory for students in different healthcare professions, including medicine, pharmacy, nursing, and social work, among other professions, in different academic years with their respective programs. This was found to both enrich the students’ attitudes towards patients with substance use disorder, and positively influence these patients’ treatment decisions [46].

Traditionally, discharging via the AMA process includes a thorough explanation of all the potential risks of leaving AMA, ensuring the patients’ understanding of these risks and their capacity to make decisions for themselves, and signing a form acknowledging these risks and accepting them [24]. Although a properly conducted discharge AMA process can provide legal protection from liability risks [47], this legal immunity does not disengage healthcare systems from their ethical commitment to minimize patients’ harm; this is also known as nonmaleficence, one of the main four Western bioethical principles [48]. Therefore, we suggest an alternative multidisciplinary approach that aims to maximize the chances of not leaving AMA or minimize the consequences of such a decision. This multidisciplinary team includes, in addition to the treating physician, a member of the patients’ experience team and a social worker.

Similarly, involving a social worker can provide multiple solutions that provide a safer discharge to the patient—for example, setting up home-based intravenous drug administration, finances, and insurance coverage. Hospitals may also provide support for patients by providing proxy care to patients’ dependents, at cost or no cost, if these patients are the sole caregivers for these dependents, which may be a good reason for these patients to leave AMA.

It thus becomes imperative to foster an ambiance of shared goals, shared knowledge and shared policies that are both universal and individualistic. In a healthcare system comprising physicians and nurses of various subspecialties, physician assistants, social workers, case managers, and nutritionists, among others, frequent communications through formal methods and policies are imperative to achieve a common goal. Thus, it becomes crucial for hospitals to possess multidisciplinary rounds that will not only develop rational coordination across disciplines but, more importantly, will speak a common language to the patient concerned [49].

Additionally, hospitals can also tackle discharge AMA by decreasing the waiting time in the emergency department (ED). Several approaches have shown efficiency in decreasing waiting times in the ED, for example, a comprehensive approach, implemented by Cambridge Hospital in Massachusetts, which included the creation of “patient partners,” a rapid assessment unit, and bedside registration, among other interventions, has led to a decrease of length of stay in the ED from 204 to 137 min, improved patients satisfaction, and decreased those who left without being seen from 4.1% to 0.9%, while accommodating a higher volume of patients [50].

#### 3.6.3. At the Policymaker Level

Perhaps the most effective way to decrease discharge AMA is through changing existing policies and/or implementing new policies. Historically, governmental policies, incentivizing or penalizing, have dramatically driven substantial changes in the medical system. For example, different healthcare policies/programs have led to improved healthcare access in non-urban areas. Another example is the change of practitioner compensation from traditional fee-for-service models to bundled-payment models that resulted in a better quality of patient experience [51]. Therefore, implementing a lower rate of discharge AMA in hospital metrics, for example, will incentivize hospitals to further invest in mitigating discharge AMA.

Furthermore, financial difficulties remain the leading cause of leaving the hospital against medical advice [11]. In a retrospective analysis of the national trauma data bank on adult trauma patients, patients with no insurance or Medicaid were more likely to leave against medical advice than patients with private insurance [52]. Therefore, policymakers should take into consideration the total expenses imposed by discharge AMA and balance that with the expenses of providing broader national insurance coverage or any form of financial support that can help decrease discharges AMA.

#### 3.6.4. Suggested Interventions to Ameliorate AMA

A number of potential interventions could be considered by healthcare teams to improve outcomes related to AMA discharge. First, rather than choosing an AMA discharge designation that could contribute to patient harm by reducing access to care, all treatment team members should work together toward a model of shared decision-making in negotiating a mutually acceptable discharge treatment plan that incorporates patients’ priorities and promotes better continuity of care in the community [37]. Ideally, a patient-centered approach would reduce the risk of a patient feeling abandoned when choosing to leave the hospital prematurely. Because nurses are often the first to identify and evaluate patients leaving AMA, they would be an integral part of this patient-centered strategy [3,20].

Similarly, recent research has demonstrated how multiple members of the healthcare team are both significantly affected by AMA discharges and are central to efforts to intervene. Derived from a patient-centered analysis of AMA discharge notes using a sociological accounts framework [49], found that physicians and nurses were both challenged by the provider-patient relationships in AMA discharge, but were also ideally situated to intervene productively [3].

## 4. Conclusions

Discharge against medical advice remains a prevalent problem, with burdens on patients’ outcomes, economy, and hospitals’ resources. It affects a wide variety of patients; however, patients with specific characteristics and comorbidities seem to be more likely to leave against medical advice. Although there are different causes for this problem, certain strategies were found to be effective in mitigating patients leaving against medical advice.

## Data Availability

No new data were created or analyzed in this study. Data sharing is not applicable to this article.
